# Age- and sex-dependent changes in levels of circulating brain-enriched microRNAs during normal aging

**DOI:** 10.18632/aging.101613

**Published:** 2018-10-31

**Authors:** Kira Sheinerman, Vladimir Tsivinsky, Aabhas Mathur, Debra Kessler, Beth Shaz, Samuil Umansky

**Affiliations:** 1 DiamiR Biosciences, Monmouth Junction, NJ 08852, USA; 2 New York Blood Center, New York, NY 10065, USA

**Keywords:** brain aging, circulating miRNAs, brain-enriched miRNAs, sex-dependent changes, aging monitoring

## Abstract

Aging is a major risk factor for many common and life-threatening pathologies. The development of reliable biomarkers of aging should lead to a better understanding of aging-associated processes and facilitate the development of therapeutic regimens that delay aging. Levels of 38 brain-enriched microRNAs (miRNA) circulating in plasma were measured by quantitative RT-PCR in two age groups: 26-35 and 56-65 years old. An miRNA-pair approach was used for data normalization and determination of effective miRNA biomarker ratios. Nineteen miRNAs, comprising miRNA pairs and pair combinations (classifiers) that effectively differentiated the age and sex (individual pairs: 74-95% and 68-95%, respectively; classifiers: up to 100% accuracy) groups, were selected for further analysis of plasma samples from 5 donor age groups: 26-35, 36-45, 46-55, 56-65 and 66-75 years old. Dynamic changes in the plasma concentrations of certain miRNAs occurred at different ages in females and males, with peaks in the 46-55-year-old and 56-65-year-old groups, respectively. This finding suggests that the changes in miRNA levels can reflect centrally regulated processes, including changes in hormone levels during menopause. Certain miRNAs and miRNA pairs correlated with age in the sex-stratified groups at different ages and should be investigated further as potentially promising biomarkers of brain aging.

## Introduction

Aging-related diseases have surpassed infectious diseases as the main cause of premature death in developed countries. Cancer, diabetes, cardiovascular diseases (CVD), Alzheimer’s (AD), Parkinson’s (PD) and other neurodegenerative diseases (NDs) are the most common aging-related pathologies. The incidence of these diseases increases rapidly with age, leading to morbidity and very often death [1]. These diseases have a highly negative economic impact on patients, their families, and society. Numerous data have demonstrated that the clinical manifestation of aging-associated diseases is preceded by prolonged (10-20 years) asymptomatic periods of pathological development [2–5]. Thus, a better understanding of the underlying processes of aging could clarify the nature of triggers involved in the initiation of these processes and the early stages of development. Despite the significant efforts made in recent years that focused on elucidating the mechanisms of aging-related disease progression, much more work is needed to develop effective assays for early detection and treatment of these diseases. Furthermore, successful treatment of one disease does not necessarily lead to significant gains in life span [6–8] because patients can die from other pathologies. As a result, a popular emerging concept is that focusing on the development of drugs targeting aging early and at its core may be more beneficial than treatment of particular diseases [9,10].

Aging has been described as “the time-dependent decline of functional capacity and stress resistance, associated with increased risk of morbidity and mortality” [11]. The data regarding the increase in longevity of various species via low-calorie diets and modulation of the IGF, sirtuin, mTOR and other pathways, as well as recent results from studies of parabiosis, indicate that a significant delay in aging is possible in principle [12,13]. Critical for developing and testing approaches to sustaining healthy living and delaying aging is the development and validation of minimally invasive, cost-effective biomarkers of aging. In addition, a quantitative definition of biomarker ranges that are characteristic of normal aging is also important for early detection of aging-related diseases. For example, synapse dysfunction and loss, ultimately followed by neuronal death, accompany normal aging [14–16]. However, rapid progression of these processes in a particular brain region could be an early indication of a neurodegenerative disease affecting this region. The same is true for other organs and tissues.

The American Federation for Aging Research [17] and the European MARK-AGE Consortium [11] have proposed several criteria for a successful biomarker of aging: (1) it must predict the rate of aging and assess where a person is in his/her lifespan better than the person’s chronological age; (2) an assay for measuring such a biomarker should be minimally invasive; and (3) the biomarker should be useful in animal models, as well as in humans, since preliminary testing of essentially all drug candidates and many therapeutic regimens is performed in non-human subjects. Traditional biomarkers of aging are based on evaluations of an individual’s general physical status, function and health of various organ systems (cardiovascular, pulmonary), cognitive function, etc. The potentially promising biomarkers of aging, which are currently being investigated, can be divided into several general groups [11–13]: (1) genetic biomarkers, including the length of telomeres in lymphocytes and other cells, age-related epigenetic changes mainly in DNA methylation, and changes in mitochondrial DNA; (2) protein-based biomarkers, including markers based on protein glycation and levels of metal-binding proteins; (3) metabolic parameters, such as hormones, lipids, and creatinine; (4) immunological and inflammatory markers, including concentrations of immunoglobulins, cytokines, and C-reactive protein in the bloodstream; (5) markers of oxidative stress; and (6) imaging biomarkers capable of registering aging-associated brain changes. To date, there is no biomarker that satisfies the three criteria listed above; some biomarker candidates are not optimal for broad clinical use because they are highly variable, invasive, laborious and/or expensive or they cannot be used in animal models.

In the current study, we assessed whether aging-associated processes in various brain regions can be detected *in vitro* via quantitative analysis of circulating brain-enriched miRNAs detectable in the bloodstream. miRNAs play important roles in the regulation of target genes by binding to complementary regions of messenger transcripts and repressing their translation or by regulating degradation [18,19]. Over 2000 miRNAs have been discovered in human cells to date, and many of these miRNAs are specific to or are overexpressed in certain organs/tissues/cells [20–23]. Some miRNAs, including those that are cell-specific, are enriched in certain cellular compartments, for example, in neurites and synapses [21–28]. Intracellular concentrations and rates of secretion of miRNAs can be dramatically affected by physiological and pathological cellular processes [29–31]. The presence of miRNAs from various organs and cell types in the blood is well documented [32–35]. Because these cell-free, circulating miRNAs can be organ-specific and are relatively stable in the blood, they are attractive biomarker candidates for various physiological and pathological processes. miRNAs appear in extracellular space and in bodily fluids due to a variety of mechanisms that remain not fully understood; these mechanisms include secretion, excretion, and blebbing [36–38]. Our studies that have been performed to date suggested that various processes, such as cell dysfunction and neurite/synapse loss, can lead to changes in miRNA concentrations in plasma, representing a rich source of potential biomarkers that detect pathology in the corresponding organ [39–44]. In addition, many publications have demonstrated that miRNA secretion, circulation in bodily fluids, and uptake by other cells are relatively common mechanisms of cell-to-cell communication, particularly in carcinogenesis, metastasis formation and other processes [45–49]. Recently, it was also demonstrated that stem cells of the hypothalamus secrete miRNAs that are transported to the CSF and potentially reach the bloodstream [50]. These miRNAs play an important role in aging-related processes. In this study, we pursue a targeted approach based on quantitative RT-PCR (qRT-PCR) analysis of a relatively small number of pre-selected miRNAs that are (1) enriched in different brain regions and (2) are present at detectable levels in plasma [21,22,51–57]. In addition, we used a miRNA-pair approach [39–44,58–61]. The concentration ratios of all miRNA pairs from the same sample were calculated, and the most promising pairs for effective differentiation of two populations or correlation with the parameter of interest, e.g. age, were selected for further testing and validation. This approach has proven to be particularly effective in the analysis of plasma concentrations of brain-enriched miRNAs to compensate not only for technical variability but also for physiological variability, e.g. changes in blood supply or blood-brain barrier permeability. Subject-to-subject variability is further decreased if a miRNA biomarker pair is comprised of two miRNAs, the plasma concentrations of which are highly correlated [39].

In this study, we evaluated the age- and sex-dependence of plasma concentrations of miRNAs enriched in different brain regions.

## RESULTS

The study was conducted in two stages. In the first set of experiments, the concentrations of 38 miRNAs (see Table 1) were measured by qRT-PCR in plasma samples from two groups of subjects: 26-35-year-olds (“young”) and 56-65-year-olds (“old”). The pre-selected set of miRNAs included brain-enriched miRNAs identified in our previous studies as potential biomarkers of neurodegenerative diseases [39,40,42] and additional miRNAs that are (i) enriched in different brain regions, neurons and glial cells, and (ii) reported in the literature and/or determined in our previous studies to be detectable in plasma. In this experiment we found miR-149, miR-154, miR-184, miR-369-3p, and miR-129-3p to be barely detectable and, hence, excluded these miRNAs from the analysis. The Cts for miR-204, miR-212, and miR-96 in many samples were higher than 36; although these data were included in the initial analysis, these miRNAs were not selected for the second set of experiments.

**Table 1:** miRNAs tested in the first study.

	**miRNA**	**Brain enrichment [****21****,****22****,****51****–****57****]**	**Present in synapses**	**Family**
**1**	**Let-7e**	Cer, MB, PG	+	miR-132
**2**	**miR-7**	PG, FC, Hip	+	
**3**	**miR-9**	FC, MB, Hip, Cer		miR-132
**4**	**miR-16**	Ubiquitous, PG		
**5**	**miR-96**	PG		
**6**	**miR-99a**	PG, MB, FC		
**7**	**miR-107**	FC, PG, Hip, MB		miR-132
**8**	**miR-127-3p**	PG, MB, FC	+	miR-134
**9**	**miR-128a**	FC, Hip, Cer	+	miR-132
**10**	**miR-129-3p**	FC, MB		
**11**	**miR-132**	PG, Hip, FC, MB	+	miR-132
**12**	**miR-134**	MB, Hip, PG	+	miR-134
**13**	**miR-135a**	PG, Hip	+	miR-132
**14**	**miR-149**	FC, MB		
**15**	**miR-153**	Hip, FC		
**16**	**miR-154**	PG, FC, MB		
**17**	**miR-181a**	MB, FC		miR-132
**18**	**miR-182**	PG		
**19**	**miR-184**	Hip, PG		
**20**	**miR-195**	PG, MB		
**21**	**miR-200a**	PG		
**22**	**miR-204**	Cer, MB, PG		
**23**	**miR-323-3p**	FC, Hip, MB	+	miR-134
**24**	**miR-335-5p**	PG, Hip		miR-132
**25**	**miR-338**	FC, Hip, MB, Cer		
**26**	**miR-370**	FC, PG	+	miR-134
**27**	**miR-369**	PG		
**28**	**miR-375**	PG		
**29**	**miR-382**	Hip, FC	+	miR-134
**30**	**miR-410**	PG, MB		miR-134
**31**	**miR-411**	PG, Hip, FC		miR-134
**32**	**miR-433**	PG, MB	+	miR-134
**33**	**miR-451**	Ubiquitous / PG, MB, FC		
**34**	**miR-485-5p**	Hip	+	miR-134
**35**	**miR-487b**	PG, FC, MB		miR-134
**36**	**miR-488**	Hip, Cer		
**37**	**miR-491-5p**	MB, FC	+	miR-132
**38**	**miR-874**	Cer, Hip	+	miR-132

Cer: cerebellum; FC: frontal cortex; Hip: hippocampus; MB: midbrain; PG: pituitary gland.

**Figure 1:**
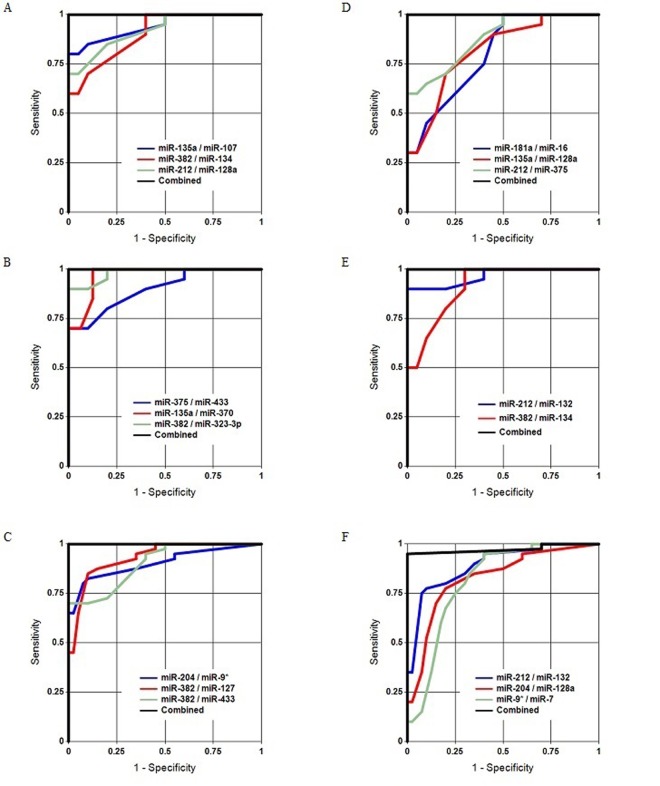
**Separation of the analyzed groups in Study 1.** (**A**, **B**, **C**) Old males vs. young males, old females vs. young females, and all old subjects vs. all young subjects, respectively. (**D**, **E**, **F**) Young females vs. young males, old females vs. old males, and all females vs. all males, respectively.

Age-related changes and sex-dependent differences in the concentrations of circulating brain-enriched miRNAs in plasma were compared as follows: (1) “young” vs. “old” males; (2) “young” vs. “old” females; (3) “young” males vs. “young” females; and (4) “old” males vs. “old” females. Although the number of samples in each group was relatively small, Figure 1, Table 2 and Table S1 demonstrate that the groups were effectively distinguished from each other by the miRNA pairs and their combinations (classifiers). These data indicated that the plasma concentrations of certain brain-enriched miRNAs are sex- and age-dependent. miRNAs comprising the most effective pairs were chosen for more detailed analyses in the larger second stage of the study. Our previous data were also considered. In particular, in Sheinerman et al. [39], the miR-134 family most effectively differentiated “young” (21-50 y.o.) and “old” (71-85 y.o.) control groups.

**Table 2:** Separation of the analyzed groups in Study 1.

Old males vs. young males					
**Pairs**	**Sens**	**Spec**	**Accur**	**AUC**	**P-Value**
**miR-135a / miR-128a**	0.90	0.90	0.90	0.99	1.20E-04
**miR-382 / miR-127**	1.00	0.70	0.85	0.99	1.20E-04
miR-212 / miR-9*	0.90	1.00	0.95	0.98	2.90E-04
miR-181a / miR-9*	1.00	0.90	0.95	0.98	2.20E-04
miR-132 / miR-9*	0.90	0.90	0.90	0.98	2.20E-04
**miR-135a / miR-129-3p**	0.84	0.84	0.84	0.97	3.80E-04
**miR-135a / miR-107**	0.86	0.86	0.86	0.97	3.80E-04
miR-99a / miR-9*	0.94	0.73	0.83	0.97	3.80E-04
**miR-212 / miR-129-3p**	0.77	0.87	0.82	0.96	3.80E-04
**miR-181a / miR-107**	0.78	0.78	0.78	0.96	8.50E-04
**miR-411 / miR-127**	0.88	0.69	0.79	0.96	6.60E-04
**miR-212 / miR-128a**	0.86	0.77	0.81	0.95	6.60E-04
**miR-382 / miR-134**	0.82	0.72	0.77	0.94	1.10E-03
**miR-135a / miR-107 + miR-382 / miR-134 + miR-212 / miR-128a**	1.00	1.00	1.00	1.00	6.70E-05

Old females vs. young females					
**Pairs**	**Sens**	**Spec**	**Accur**	**AUC**	**P-Value**
**miR-382 / miR-323-3p**	0.60	1.00	0.80	0.99	1.20E-04
**miR-99a / miR-370**	0.70	1.00	0.83	0.98	4.20E-04
**miR-195 / miR-16**	0.90	0.90	0.90	0.98	2.90E-04
**miR-135a / miR-370**	0.70	1.00	0.83	0.98	4.20E-04
**miR-382 / miR-127**	0.90	0.90	0.90	0.98	2.20E-04
**miR-382 / miR-433**	0.90	0.80	0.85	0.98	2.20E-04
**miR-99a / miR-433**	0.80	0.90	0.85	0.96	8.50E-04
**miR-99a / miR-154**	0.62	0.90	0.74	0.96	1.10E-03
**miR-181a / miR-370**	1.00	0.75	0.89	0.96	7.80E-04
miR-181a / miR-9*	0.86	0.86	0.86	0.96	5.00E-04
**miR-181a / miR-491**	0.80	0.90	0.85	0.96	6.60E-04
**miR-382 / miR-370**	0.88	0.73	0.81	0.96	1.10E-03
**miR-375 / miR-433**	0.80	0.80	0.80	0.94	1.10E-03
**miR-375 / miR-433 + miR-135a / miR-370 + miR-382 / miR-323-3p**	1.00	1.00	1.00	1.00	6.70E-05

All old subjects vs. all young subjects					
**Pairs**	**Sens**	**Spec**	**Accur**	**AUC**	**P-Value**
miR-181a / miR-9*	1.00	0.85	0.93	0.96	6.00E-07
miR-135a / miR-9*	0.85	1.00	0.93	0.95	2.30E-06
**miR-382 / miR-127**	0.88	0.83	0.86	0.95	1.30E-06
**miR-382 / miR-134**	0.86	0.81	0.84	0.95	1.70E-06
**miR-382 / miR-323-3p**	0.82	0.82	0.82	0.94	2.90E-06
miR-99a / miR-9*	0.80	0.80	0.80	0.92	7.00E-06
miR-204 / miR-9*	0.84	0.84	0.84	0.92	9.00E-06
**miR-181a / miR-107**	0.75	0.80	0.78	0.91	1.60E-05
**miR-382 / miR-433**	0.84	0.69	0.76	0.91	7.00E-06
**miR-135a / miR-128a**	0.80	0.70	0.75	0.89	5.80E-05
**miR-487b / miR-127**	0.75	0.75	0.75	0.88	4.60E-05
**miR-135a / miR-338-3p**	0.79	0.74	0.77	0.87	1.50E-04
**miR-99a / miR-338-3p**	0.80	0.65	0.73	0.87	1.40E-04
miR-204 / miR-9* + miR-382 / miR-127 + miR-382 / miR-323-3p	1.00	1.00	1.00	1.00	2.90E-08

Young females vs. young males					
**Pairs / Combos**	**Sens**	**Spec**	**Accur**	**AUC**	**P-Value**
**miR-212 / miR-874**	0.86	0.86	0.86	0.97	2.90E-04
**miR-212 / miR-7**	0.82	0.93	0.88	0.95	8.50E-04
**miR-212 / miR-195**	0.83	0.73	0.78	0.94	1.40E-03
**miR-212 / miR-128a**	0.79	0.69	0.74	0.93	2.30E-03
**miR-212 / miR-375**	0.80	0.70	0.75	0.92	1.80E-03
**miR-212 / miR-16**	0.77	0.68	0.73	0.90	2.90E-03
**miR-204 / miR-128a**	0.78	0.78	0.78	0.90	2.90E-03
**miR-135a / miR-128a**	0.74	0.74	0.74	0.89	5.70E-03
**miR-212 / miR-184**	0.67	0.74	0.70	0.89	1.70E-02
**miR-411 / miR-323-3p**	0.76	0.76	0.76	0.89	7.00E-03
**miR-181a / miR-107**	0.62	0.73	0.68	0.88	7.00E-03
**miR-212 / miR-182**	0.80	0.70	0.75	0.88	5.70E-03
**miR-212 / miR-491**	0.80	0.60	0.70	0.88	4.60E-03
**miR-181a / miR-16 + miR-135a / miR-128a + miR-212 / miR-375**	1.00	1.00	1.00	1.00	6.70E-05

Old females vs. old males					
**Pairs / Combos**	**Sens**	**Spec**	**Accur**	**AUC**	**P-Value**
**miR-212 / miR-132**	0.90	1.00	0.95	0.98	2.20E-04
**miR-375 / miR-7**	0.90	0.80	0.85	0.97	3.80E-04
**miR-200a / miR-7**	0.90	0.90	0.90	0.97	5.00E-04
**miR-204 / miR-7**	0.77	0.86	0.82	0.96	5.00E-04
**miR-204 / let-7e**	0.84	0.73	0.79	0.95	6.60E-04
**miR-195 / miR-7**	0.74	0.84	0.79	0.95	8.50E-04
**miR-382 / miR-134**	0.85	0.75	0.80	0.95	8.50E-04
**miR-200a / let-7e**	0.71	0.80	0.76	0.94	1.80E-03
miR-9* / miR-7	0.83	0.83	0.83	0.94	8.50E-04
miR-9* / miR-135a	0.80	0.70	0.75	0.94	1.40E-03
**miR-212 / miR-7**	0.80	0.80	0.80	0.94	8.50E-04
**miR-181a / miR-7**	0.82	0.72	0.77	0.94	8.50E-04
**miR-874 / miR-7**	0.90	0.70	0.80	0.94	6.60E-04
**miR-212 / miR-132 + miR-382 / miR-134**	1.00	1.00	1.00	1.00	6.70E-05

All females vs. all males					
**Pairs / Combos**	**SENS**	**SPEC**	**ACCUR**	**AUC**	**P-Value**
**miR-212 / miR-7**	0.84	0.84	0.84	0.93	8.00E-06
**miR-212 / miR-132**	0.82	0.77	0.79	0.92	1.10E-05
**miR-212 / miR-16**	0.82	0.77	0.79	0.89	6.40E-05
**miR-204 / miR-128a**	0.76	0.81	0.78	0.86	2.10E-04
**miR-874 / miR-7**	0.67	0.77	0.72	0.85	2.80E-04
**miR-212 / miR-107**	0.73	0.68	0.71	0.84	4.60E-04
**miR-212 / miR-128a**	0.70	0.70	0.70	0.84	5.10E-04
**miR-212 / miR-195**	0.64	0.79	0.72	0.84	4.60E-04
**miR-204 / miR-7**	0.82	0.56	0.69	0.84	2.80E-04
miR-9* / miR-7	0.75	0.75	0.75	0.84	3.80E-04
**miR-181a / miR-7**	0.62	0.72	0.67	0.84	2.50E-04
**miR-212 / miR-874**	0.69	0.69	0.69	0.83	7.40E-04
**miR-204 / miR-107**	0.71	0.76	0.74	0.83	8.10E-04
miR-212 / miR-195 + miR-204 / miR-128a + miR-9* / miR-7	0.85	0.95	0.90	0.97	5.20E-07

**Figure 2:**
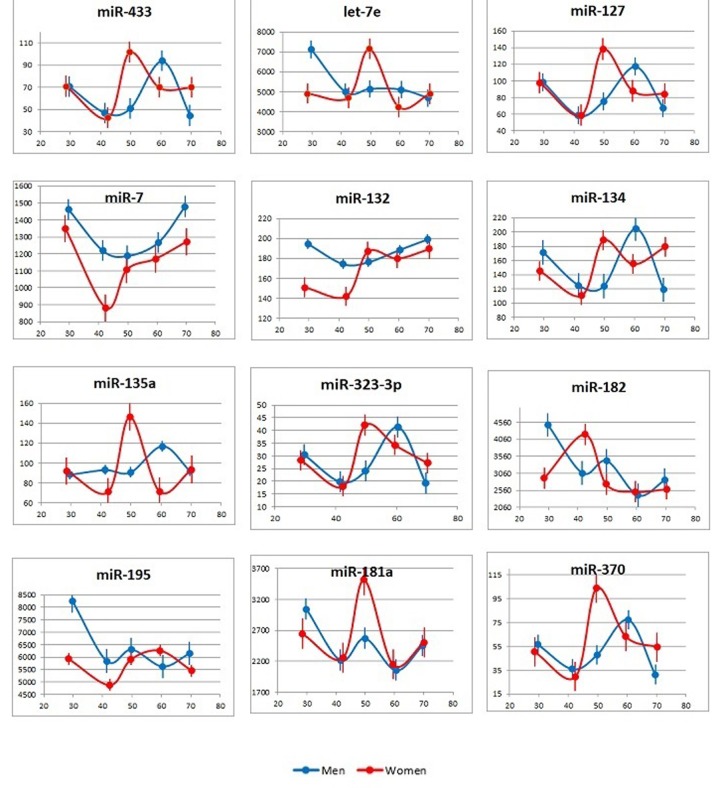
**Age-dependent changes in plasma concentrations of the tested brain-enriched miRNAs.** Data are presented as the average and standard deviation for each age group.  X axis: age; Y axis: number of miRNA copies per 1 μl of plasma.

Nineteen miRNAs (see Table S1) were selected for the analysis of the plasma samples from 100 subjects: 5 groups, namely, 26-35, 36-45, 46-55, 56-65 and 66-75-year-olds, with 10 females and 10 males in each group. Figure 2 presents the age-dependent changes in the plasma concentrations of individual miRNAs in females and males (averages for 10 subjects in each group). Several observations are of interest here: (1) among the tested miRNAs, the concentration of no single miRNA correlated with age in all (female and male) subjects; (2) the age-dependent changes in miRNA concentrations were different in male and female subjects; (3) the age-dependent changes in the concentrations of some miRNAs, e.g. members of the miR-134 family, were similar across the sex-stratified groups; and (4) there were peaks in the plasma concentrations of many miRNAs in the 46-55-year-old females and the 56-65-years-old males.

**Figure 3:**
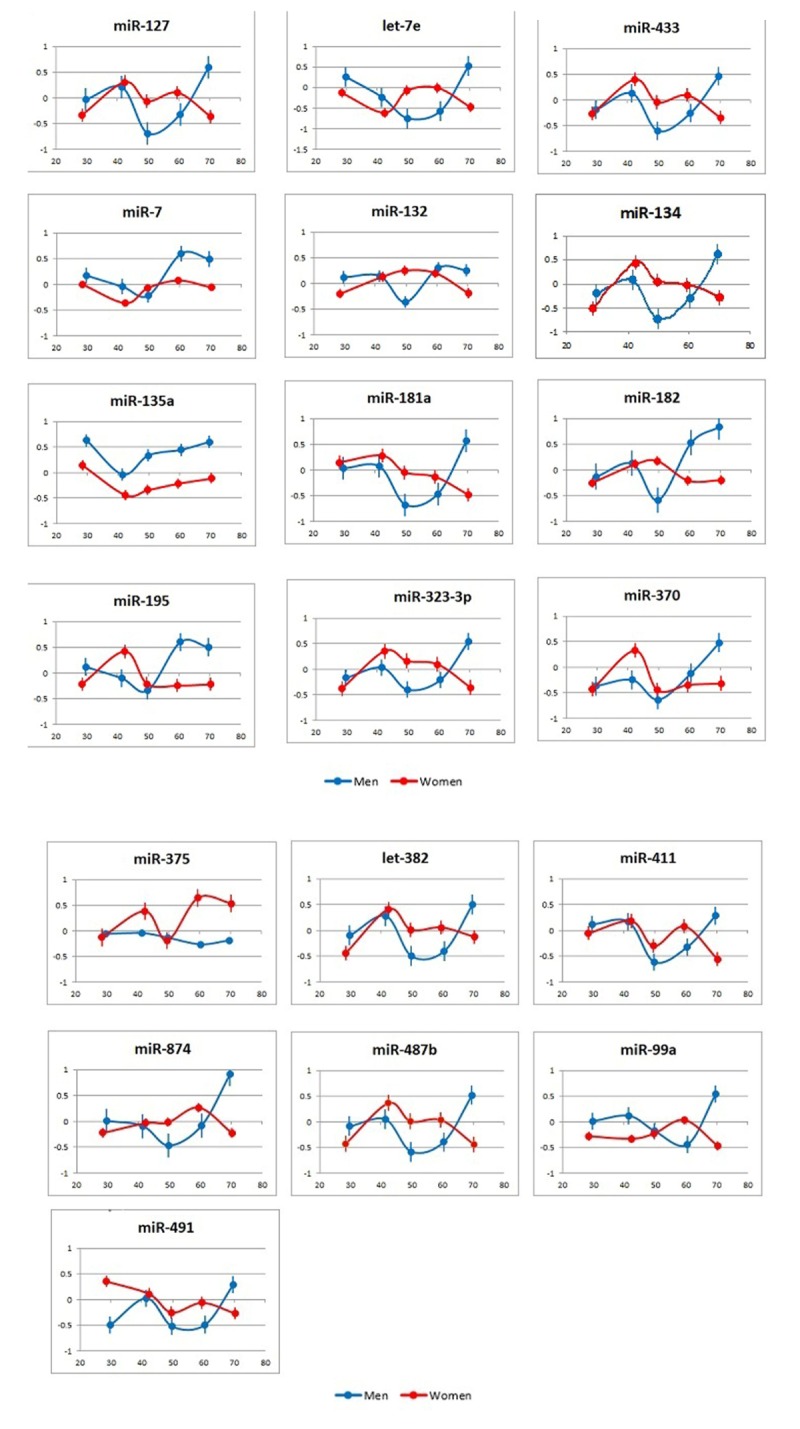
**Correlation of miRNA plasma concentrations with age in male and female subjects.** Data are presented as average and standard deviation for each age cohort.  X axis: age; Y axis: correlation of miRNA plasma concentrations with subject age in the analyzed groups (r).

One key finding of the study was that, among the tested miRNAs, no single one could be used as an age biomarker for the entire tested age continuum. More detailed analyses also revealed that there was no miRNA pair formed by the tested miRNAs that correlated with a subject’s age over a prolonged time period. We further analyzed the correlations of individual miRNAs with age across 10-year spans in the sex-stratified groups (Figure 3 and Figure S1 and Table S2). As expected, from the dynamics of miRNA plasma concentrations (Figure 2), the age spans, during which correlations between the levels of certain individual miRNAs and subject age are observed, were significantly different for female and male subjects. Further, although the division of age groups was done arbitrarily by 10-year spans, and additional studies are needed to assess the physiological relevance of the present findings, the age groups were effectively separated from each other by multiple miRNA pairs and their combinations (Figure S2, Tables 3).

**Table 3:** Males. miRNA pairs and their combinations that differentiated consecutively aged males from each other.

**M_36/M_26 group**					
**Pairs / Combos**	**SENS**	**SPEC**	**ACCUR**	**AUC**	**P-Value**
**miR-135a / let-7e**	0.77	0.86	0.82	0.94	1.40E-03
**miR-135a / miR-487b**	0.86	0.76	0.81	0.9	4.60E-03
**miR-132 / miR-411**	0.67	0.9	0.79	0.89	1.00E-02
**miR-132 / miR-127**	0.76	0.76	0.76	0.83	1.90E-02
**miR-382 / miR-487b**	0.76	0.76	0.76	0.87	1.60E-02
**miR-135a / miR-411**	0.78	0.7	0.74	0.87	1.20E-02
**miR-135a / miR-127**	0.82	0.64	0.73	0.86	8.60E-03
**miR-132 / miR-487b**	0.77	0.68	0.73	0.82	1.90E-02
**miR-134 / miR-127**	0.77	0.68	0.73	0.83	2.30E-02
**miR-135a / miR-134**	0.72	0.72	0.72	0.86	1.60E-02
**miR-135a / miR-181a**	0.8	0.6	0.7	0.84	1.90E-02
**miR-99a / miR-487b**	0.58	0.78	0.68	0.81	2.70E-02
**miR-99a / miR-127**	0.58	0.77	0.68	0.81	2.70E-02
**miR-135a / miR-382**	0.67	0.67	0.67	0.82	1.90E-02
**miR-135a / miR-7**	0.77	0.58	0.67	0.83	4.40E-02
**miR-134 / miR-487b**	0.57	0.76	0.66	0.8	2.70E-02
**miR-135a / miR-370**	0.88	0.44	0.65	0.81	3.80E-02
**miR-491-5p / miR-411**	0.74	0.57	0.65	0.8	4.70E-02
**miR-874 / miR-487b**	0.6	0.7	0.65	0.81	4.40E-02
**miR-135a / miR-433**	0.67	0.6	0.63	0.8	4.00E-02
**miR-135a /let-7e + miR-132 / miR-411**	0.90	0.80	0.85	0.95	8.50E-04

**M_46 / M36**					
**Pairs / Combos**	**SENS**	**SPEC**	**ACCUR**	**AUC**	**P-Value**
**miR-370 / miR-134**	0.78	0.78	0.78	0.91	6.60E-03
**miR-127 / miR-135a**	0.77	0.67	0.72	0.83	2.30E-02
**miR-127 / miR-134**	0.66	0.76	0.71	0.8	2.70E-02
**miR-134 / miR-135a**	0.62	0.73	0.68	0.83	4.40E-02
**miR-323-3p / miR-135a**	0.8	0.5	0.65	0.82	3.20E-02
**miR-7 / miR-135a**	0.63	0.63	63.00	0.78	1.10E-02
**miR-487b / miR-134**	0.66	0.56	0.61	0.78	8.10E-02
**miR-7 / miR-135a + miR-127 / miR-134 + miR-487b / miR-134**	0.90	0.80	0.85	0.92	3.60E-03

**M_56 / M46**					
**Pairs / Combos**	**SENS**	**SPEC**	**ACCUR**	**AUC**	**P-Value**
**miR-433 / miR-182**	0.88	0.7	0.78	0.84	1.80E-02
**miR-433 / miR-411**	0.75	0.8	0.78	0.91	4.30E-03
**miR-433 / miR-132**	0.78	0.73	0.75	0.81	5.00E-02
**miR-382 / miR-411**	0.72	0.76	0.74	0.86	1.50E-02
**miR-433 / miR-195**	0.63	0.8	0.72	0.86	3.40E-02
**miR-491-5p / miR-181a**	0.7	0.7	0.7	0.81	3.20E-02
**miR-135a / miR-182**	0.59	0.79	0.69	0.84	1.60E-02
**miR-491-5p / let-7e**	0.74	0.63	0.69	0.81	3.20E-02
**miR-433 / miR-181a**	0.71	0.66	0.68	0.83	2.80E-02
**miR-135a / miR-181a**	0.62	0.73	0.68	0.82	2.70E-02
**miR-370 / miR-182**	0.48	0.83	0.67	0.85	2.80E-02
**miR-487b / miR-411**	0.59	0.73	0.67	0.89	2.30E-02
**miR-135a / miR-7**	0.49	0.78	0.64	0.81	2.70E-02
**miR-433 / miR-370**	0.47	0.68	0.58	0.8	4.60E-02
**miR-433 / miR-411 + miR-433 / miR-370 + miR-370 / miR-182**	0.88	0.90	0.89	0.96	6.60E-04

**M_66 / M56**					
**Pairs / Combos**	**SENS**	**SPEC**	**ACCUR**	**AUC**	**P-Value**
**miR-182 / miR-370**	0.69	0.83	0.76	0.93	7.50E-03
**miR-134 / miR-382**	0.8	0.67	0.74	0.84	1.50E-02
**miR-7 / miR-370**	0.71	0.71	0.71	0.87	2.00E-02
**miR-195 / miR-370**	0.78	0.65	0.71	0.87	2.00E-02
**miR-411 / miR-382**	0.67	0.75	0.71	0.84	2.40E-02
**miR-182 / miR-433**	0.58	0.85	0.7	0.9	7.10E-03
**miR-195 / miR-433**	0.63	0.79	0.7	0.84	3.40E-02
**miR-874 / miR-433**	0.84	0.53	0.7	0.84	2.80E-02
**miR-181a / miR-370**	0.63	0.76	0.69	0.88	2.70E-02
**miR-874 / miR-370**	0.77	0.62	0.69	0.82	4.80E-02
**miR-132 / miR-433**	0.56	0.82	0.68	0.86	1.50E-02
**miR-127 / miR-370**	0.6	0.75	0.68	0.82	4.80E-02
**miR-181a / miR-433**	0.6	0.75	0.67	0.83	2.80E-02
**miR-7 / miR-433**	0.6	0.75	0.67	0.83	2.30E-02
**miR-134 / miR-370**	0.6	0.74	0.67	0.85	3.70E-02
**miR-99a / miR-382**	0.66	0.66	0.66	0.83	1.90E-02
**miR-181a / miR-874**	0.66	0.66	0.66	0.8	3.80E-02
**miR-182 / miR-411**	0.52	0.82	0.66	0.86	1.90E-02
**miR-127 / miR-382**	0.51	0.81	0.66	0.85	1.10E-02
**miR-181a / let-7e**	0.7	0.6	0.65	0.81	3.20E-02
**miR-132 / miR-370**	0.57	0.71	0.64	0.87	2.70E-02
**miR-182 / miR-433 + miR-127 / miR-382 + miR-134 / miR-370**	0.90	1.00	0.95	0.98	2.20E-04

SENS: sensitivity; SPEC: specificity; ACCUR: accuracy; AUC: area under the ROC curve; M: males; F: females. Numbers indicate the youngest age of each respective group (e.g. F_26 is the female 26-35-year-old group).

**Table 3:** Females. miRNA pairs and their combinations that differentiated consecutively aged females from each other.

**F_36 / F_26**					
**Pairs / Combos**	**SENS**	**SPEC**	**ACCUR**	**AUC**	**P-Value**
**miR-182 / miR-375**	0.76	0.76	0.76	0.83	2.30E-02
**miR-487b / miR-370**	0.8	0.73	0.76	0.87	2.20E-02
**miR-134 / miR-370**	0.68	0.76	0.73	0.83	2.90E-02
**miR-132 / miR-375**	0.67	0.77	0.72	0.83	2.30E-02
**miR-874 / miR-375**	0.72	0.72	0.72	0.85	2.30E-02
**miR-99a / miR-375**	0.67	0.75	0.71	0.92	4.60E-03
**let-7e / miR-375**	0.71	0.71	0.71	0.85	2.30E-02
**miR-134 / miR-127**	0.7	0.7	0.7	0.81	2.70E-02
**miR-433 / miR-370**	0.52	0.8	0.69	0.87	2.00E-02
**miR-182 / miR-382**	0.54	0.78	0.67	0.82	3.30E-02
**miR-874 / miR-7**	0.54	0.64	0.59	0.8	2.30E-02
**miR-135a / miR-7**	0.42	0.74	0.58	0.8	3.80E-02
**let-7e / miR-375 + miR-134 / miR-127 + miR-487b / miR-370**	0.90	0.80	0.85	0.96	6.60E-04

**F_46 / F_36**					
**Pairs / Combos**	**SENS**	**SPEC**	**ACCUR**	**AUC**	**P-Value**
**miR-370 / miR-323-3p**	0.68	0.82	0.75	0.87	2.00E-02
**miR-491-5p / miR-182**	0.73	0.73	0.73	0.86	8.60E-03
**miR-375 / miR-99a**	0.83	0.62	0.73	0.82	3.20E-02
**miR-411 / miR-182**	0.8	0.63	0.72	0.86	9.10E-03
**miR-370 / miR-382**	0.66	0.77	0.71	0.89	1.90E-02
**miR-370 / miR-134**	0.71	0.71	0.71	0.87	2.00E-02
**miR-370 / miR-127**	0.71	0.71	0.71	0.92	1.50E-02
**miR-135a / miR-182**	0.71	0.71	0.71	0.83	2.30E-02
**miR-491-5p / miR-874**	0.81	0.61	0.71	0.86	1.60E-02
**miR-375 / miR-182**	0.66	0.76	0.71	0.83	1.60E-02
**miR-370 / miR-487b**	0.64	0.77	0.7	0.85	3.70E-02
**miR-132 / miR-874**	0.54	0.86	0.7	0.87	7.00E-03
**miR-370 / miR-182**	0.62	0.77	0.69	0.83	3.70E-02
**miR-127 / miR-182**	0.68	0.68	0.68	0.81	2.30E-02
**miR-135a / miR-874**	0.53	0.84	0.68	0.85	1.10E-02
**miR-195 / miR-182**	0.67	0.67	0.67	0.83	1.90E-02
**miR-370 / miR-874**	0.66	0.66	0.66	0.84	4.80E-02
**miR-433 / miR-182**	0.57	0.76	0.66	0.82	4.00E-02
**miR-411 / miR-134**	0.86	0.4	0.65	0.81	3.40E-02
**miR-375 / miR-874**	0.5	0.8	0.65	0.86	1.30E-02
**miR-411 / miR-182 + miR-135a / miR-874 + miR-375 / miR-99a**	0.9	0.9	0.9	0.99	1.60E-04

**F_56 / F_46**					
**Pairs / Combos**	**SENS**	**SPEC**	**ACCUR**	**AUC**	**P-Value**
**miR-491-5p / miR-411**	0.73	0.73	0.73	0.82	3.20E-02
**miR-195 / miR-135a**	0.8	0.6	0.7	0.86	8.60E-03
**miR-195 / miR-99a**	0.74	0.63	0.69	0.86	8.60E-03
**miR-182 / miR-135a**	0.63	0.74	0.69	0.83	1.90E-02
**miR-323-3p / miR-411**	0.74	0.63	0.68	0.82	1.90E-02
**miR-382 / miR-411**	0.74	0.63	0.68	0.82	2.70E-02
**miR-132 / miR-135a**	0.67	0.67	0.67	0.87	2.30E-02
**miR-132 / let-7e**	0.66	0.66	0.66	0.81	4.40E-02
**miR-195 / miR-411**	0.8	0.5	0.65	0.83	2.30E-02
**miR-132 / miR-411**	0.79	0.49	0.64	0.84	3.20E-02
**miR-323-3p / miR-370**	0.57	0.71	0.64	0.83	4.80E-02
**miR-195 / miR-135a + miR-195 / miR-99a + miR-382 / miR-411**	1.00	0.90	0.95	0.99	1.60E-04

**F_66 / F_56**					
**Pairs / Combos**	**SENS**	**SPEC**	**ACCUR**	**AUC**	**P-Value**
**miR-135a / miR-195**	0.7	0.7	0.7	0.83	1.30E-02
**miR-181a / miR-195**	0.69	0.69	0.69	0.84	1.90E-02
**miR-134 / miR-323-3p**	0.69	0.62	0.66	0.8	3.30E-02
**miR-874 / miR-195**	0.66	0.66	0.66	0.82	3.20E-02
**miR-134 / miR-382**	0.77	0.54	0.63	0.8	4.40E-02
**miR-134 / miR-323-3p + miR-135a / miR-195 + miR-874 / miR-195**	0.80	0.80	0.80	0.92	1.80E-03

SENS: sensitivity; SPEC: specificity; ACCUR: accuracy; AUC: area under the ROC curve; M: males; F: females. Numbers indicate the youngest age of each respective group (e.g. F_26 is the female 26-35-year-old group).

miRNA pairs whose correlations with age, particularly in the sex-stratified groups, were found to be statistically significant are presented in Figure S3 and Table 4.

**Table 4:** Spearman correlations of the miRNA pair combinations with subject ages in each of the 10 groups.

	**Female**				**Male**			
**Age Range**	**Pairs / Combos**	**Corr**	**RSD**	**P-Val**	**Pairs / Combos**	**Corr**	**RSD**	**P-Val**
**26-35**	**miR-135a / miR-323-3p**	0.57	0.96	0.04	**miR-135a / miR-491-5p**	0.77	1.90	<0.01
	**miR-411 / miR-370**	0.65	0.88	0.02	**miR-135a / miR-195**	0.64	2.27	0.02
	**miR-411 / miR-127**	0.58	0.95	0.04	**miR-411 / miR-323-3p**	0.70	2.10	0.01
					**miR-127 / miR-323-3p**	0.71	2.08	0.01
	**miR-135a / miR-323-3p + miR-411 / miR-370 + miR-411 / miR-127**	0.77	0.74	<0.01	**miR-135a / miR-491-5p + miR-135a / miR-195 + miR-411 / miR-323-3p + miR-127 / miR-323-3p**	0.95	0.96	<0.01
**36-45**	**miR-134 / miR-135a**	0.59	2.73	0.04	**miR-127 / miR-134**	0.53	1.66	0.06
	**miR-375 / let-7e**	0.61	2.70	0.03	**miR-382 / let-7e**	0.59	1.57	0.04
	**miR-375 / miR-135a**	0.60	2.70	0.03	**miR-132 / let-7e**	0.53	1.65	0.06
	**miR-134 / miR-135a + miR-375 / let-7e + miR-375 / miR-135a**	0.91	1.42	<0.01	**miR-127 / miR-134 + miR-382 / let-7e + miR-132 / let-7e**	0.73	1.34	<0.01
**46-55**	**miR-182 / miR-195**	0.58	2.04	0.04	**miR-135a / miR-99a**	0.73	2.23	0.01
	**miR-433 / miR-411**	0.74	1.68	<0.01	**miR-323-3p / miR-127**	0.78	2.05	<0.01
	**let-7e / miR-135a**	0.53	2.13	0.06	**miR-181a / miR-411**	0.73	2.24	<0.01
	**miR-182 / miR-195 + miR-433 / miR-411 + let-7e / miR-135a**	0.85	1.34	<0.01	**miR-135a / miR-99a + miR-323-3p / miR-127 + miR-181a / miR-411**	0.93	1.24	<0.01
**56-65**	**miR-323-3p / miR-433**	0.64	1.69	0.02	**miR-182 / miR-491-5p**	0.70	2.82	0.01
	**miR-382 / miR-134**	0.65	1.68	0.02	**miR-135a / miR-99a**	0.74	2.65	<0.01
	**miR-132 / miR-135a**	0.70	1.58	0.01				
	**miR-323-3p / miR-433 + miR-382 / miR-134 + miR-132 / miR-135a**	0.93	0.79	<0.01	**miR-182 / miR-491-5p + miR-135a / miR-99a**	0.73	1.95	<0.01
**66-75**	**miR-132 / miR-181a**	0.69	2.16	0.01	**miR-874 / miR-491-5p**	0.68	2.08	0.02
					**miR-874 / miR-132**	0.92	1.57	<0.01
	**miR-127 / miR-487b**	0.70	2.14	0.01	**miR-127 / miR-433**	0.67	2.11	0.02
	**miR-132 / miR-181a + miR-127 / miR-487b**	0.76	1.94	<0.01	**miR-874 / miR-132 + miR-874 / miR-491-5p + miR-127 / miR-433**	0.84	1.53	<0.01

Corr: correlation; RSD: residual standard deviation; P-Val: P-value.

These data demonstrated that miRNA pairs and classifiers of brain-enriched miRNAs circulating in plasma can be potentially used as aging biomarkers during specific age spans. Larger studies are needed to validate these findings and to better define the specific age spans.

## DISCUSSION

The data obtained in this feasibility study demonstrated the potential use of circulating brain-enriched miRNAs as biomarkers of brain aging. Although we did not find a brain-enriched miRNA (or a miRNA pair) whose levels in plasma correlated with the wide age range of 26-75 years, we established miRNA pairs that correlated with age in sex-stratified groups covering 10-year spans. Larger studies are needed to better define the exact age spans when the miRNA levels change.

Age-associated changes in plasma concentrations of the brain-enriched miRNAs tested in this study are likely reflective of molecular and physiological processes in the brain, such as the following: (i) miRNA expression; (ii) miRNA secretion/excretion (this possibility is discussed in the accompanying paper [62]); (iii) rate of synapse dysfunction and loss, especially in older subjects; (iv) neuronal death; (v) blood supply; and (vi) blood-brain barrier permeability. Substantially identical and overlapping patterns of decreases and increases in plasma levels of multiple brain-enriched miRNAs indirectly indicated that these are centrally regulated phenomena. The different dynamics in the plasma concentrations of brain-enriched miRNAs in female and male subjects, which were particularly prominent in the 46-65-year-old group, coincided with the changes in sex hormone levels. Maximum levels of miR-134 family members and certain other miRNAs in the plasma of female subjects were reached in the 46-55-year-old group. Interestingly, this result corresponds to perimenopause and menopause in women, when a significant drop in circulating estradiol occurs. In males, peaks in the miRNA concentrations were reached in the 56-65-year-old group, possibly reflecting slower changes in testosterone decreases. Thus, one can hypothesize that sex hormones modulate miRNA synthesis and/or secretion. This concept is in agreement with the recently reported inhibition of members of the miR-134 family (miR-127, miR-134, miR-370, miR-432) and other miRNAs by estradiol in the neonatal hypothalamus [63]. The miRNA biomarker candidates established in this study should be further evaluated alongside other molecular biomarkers of aging, such as telomerase length shortening and DNA methylation [11–13]. Larger studies, including longitudinal ones, will be necessary for determining the use of miRNA biomarker classifiers in clinical research. Further, we propose testing other circulating organ-enriched miRNAs as biomarkers of aging in respective organs and tissues. As was recently demonstrated [41–43], significant changes in the normal values of such biomarkers can signal more serious pathologic processes than aging alone.

## METHODS

### Subjects and plasma collection

All subjects in the study were blood donors at the New York Blood Center who were without known neurodegenerative or neurological conditions. Two sets of blood collection were performed: 1) 40 subjects, 26-35 years of age (10 “young females” and 10 “young males”) and 56–65 years of age (10 “old females” and 10 “old males”); and 2) 100 subjects, 26-35, 36-45, 46-55, 56-65 and 66-75 years of age, with 10 females and 10 males in each age group.

Samples for the study were collected from blood donors at the New York Blood Center. Blood was collected in 6-ml lavender-top K_2_EDTA tubes and then centrifuged at 4^o^C at 2,000xg. Plasma was aliquoted into RNase-free, 2 ml round-bottom microcentrifuge tubes (Biotix, San Diego, CA and frozen at -80^o^C within 2 hours of the blood collection. The demographic characteristics of the study groups are summarized in Table 5.

**Table 5:** Age groups of the normal subjects analyzed in both studies.

**Sex**	**Age (y.o.)**	**Study**	
		**1st**	**2nd**
**Male**	26-35	10	10
	36-45	-	10
	46-55	-	10
	56-65	10	10
	66-75	-	10
**Female**	26-35	10	10
	36-45	-	10
	46-55	-	10
	56-65	10	10
	66-75	-	10

### Plasma RNA purification and qRT-PCR miRNA analysis

miRNA isolation and qRT-PCR analysis were performed in accordance with the following protocol (Asuragen, Austin, TX). RNA was extracted from 1 ml of plasma using a TRIzol treatment and silica (Ambion Glass Fiber Microcolumn)-binding protocol (http://asuragen.com/wp-content/uploads/2016/05/biomarkers.pdf). Single-target qRT-PCR was performed using the TaqMan Reverse Transcription Kit and miRNA-specific stem-loop primers (Thermo Fisher). QC of miRNA preps was performed by testing two ubiquitous miRNAs in each plasma prep; all samples with values within two standard deviations of the average value qualified as acceptable for analysis. miRNAs with cycle thresholds (Ct)>37 were excluded from the analysis of each respective sample. The RT step for generation of cDNA from selected miRNAs was performed in triplicate using miRNA-specific primers, and 2-µl plasma equivalents were present in the final PCR. Calibration curves for each miRNA were generated to calculate the miRNA concentration in copy numbers.

### Statistical methods

All statistical calculations were performed through the use of custom software developed at DiamiR [39]. The application was designed in .NET technology using a set of .NET statistical packages. Mann-Whitney U-tests were used to evaluate the significance of the differences between the two groups of subjects in the various miRNA pairs. Receiver operating characteristic (ROC) curves were constructed, and the area under the ROC curves (AUC), sensitivity, specificity, and accuracy of the miRNA pairs and their combinations were calculated. To reduce instrumental errors, calibration curves for each miRNA were generated using synthetic miRNAs. Average miRNA concentrations and correlations between individual miRNAs or miRNA pairs and age were calculated using copy numbers. Effective pair combinations (miRNA classifiers) were defined using logistic regression. The residual standard deviation (RSD) of the linear regression was used to estimate the age prediction power of the miRNA biomarker pairs. Effective pair combinations that correlated with age were created using pair data averaging.

## Supplementary Material

Supplementary Figures

Supplementary Tables
