# Supplementary material for: Age- and sex-dependent changes in levels of circulating brain-enriched microRNAs during normal aging
Source: Aging (Albany NY). 2018 Oct 31;10(10):3017–41. doi: 10.18632/aging.101613 (PMC6224262; doi:10.18632/aging.101613)
Supplement: Supplementary Figures [file aging-10-101613-s001.pdf]

SUPPLEMENTARY FIGURES

A

B

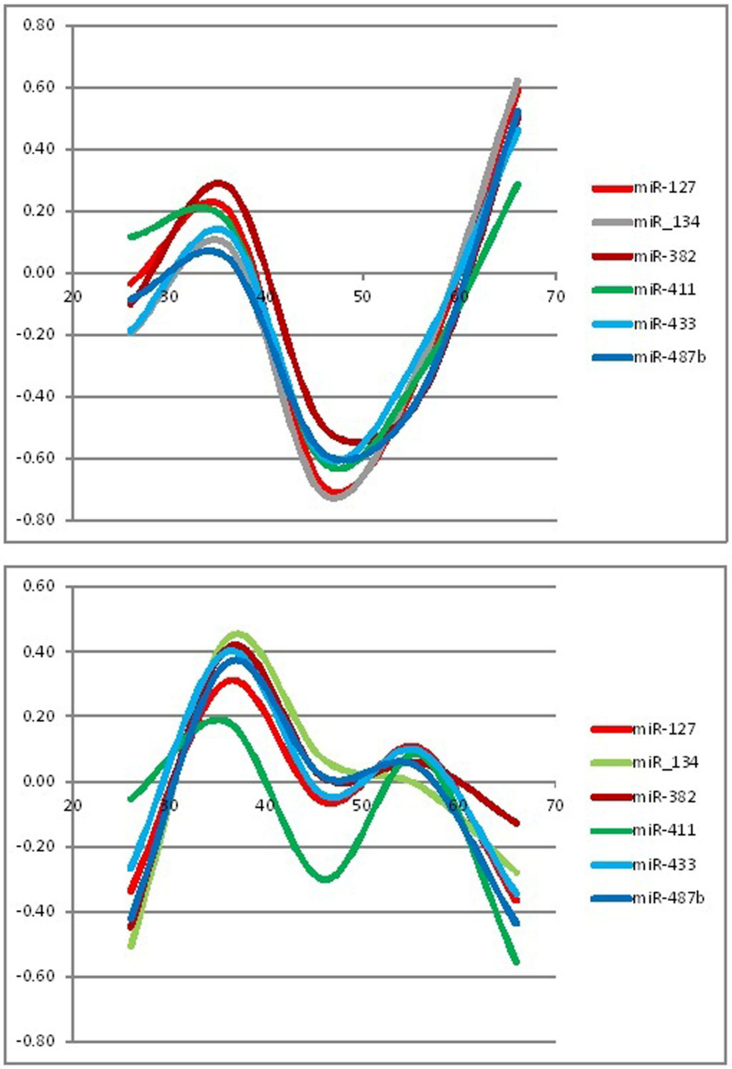

Figure S1. Correlation of miR-134 family plasma concentrations with age in the male (**A**) and female (**B**) subjects. X axis: age; Y axis: correlation of miRNA plasma concentrations with subject age in the analyzed groups (r).

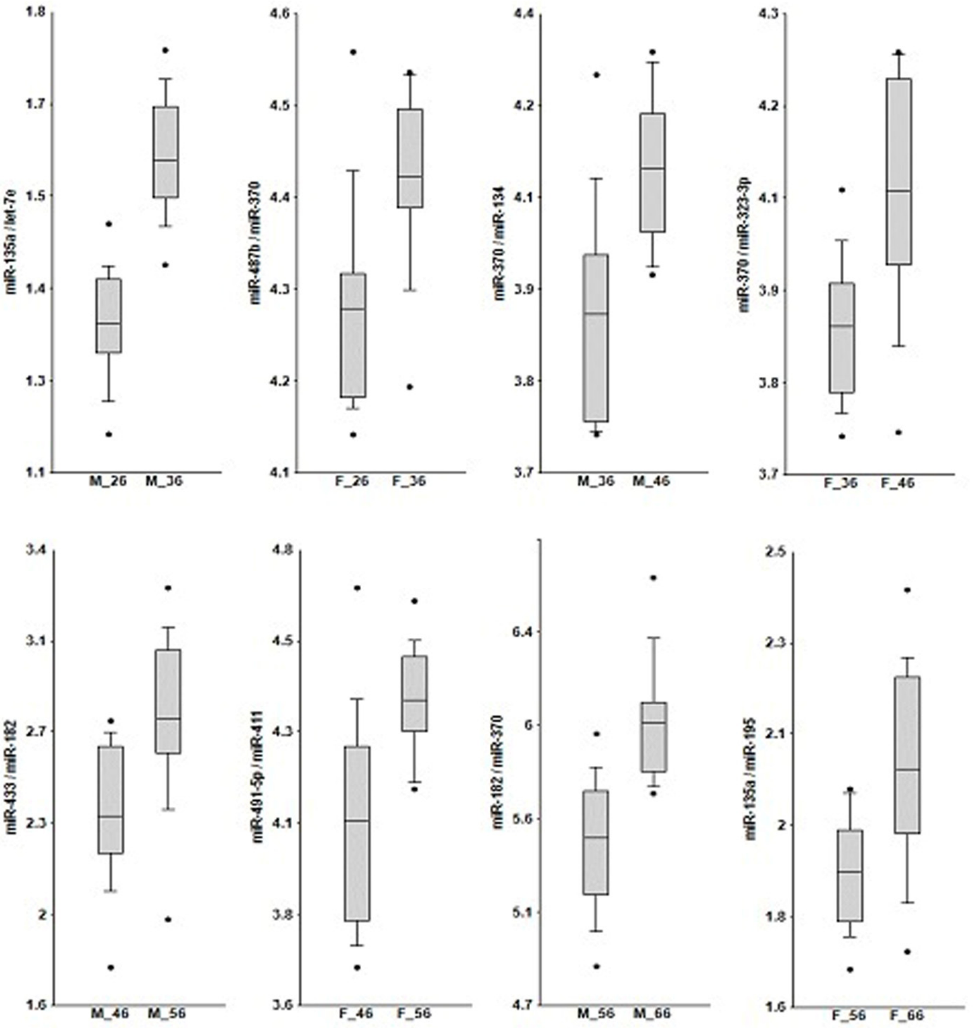

Figure S2. Differentiation of consecutively aged male or female groups from each other by select microRNA pairs. M: males; F: females. Numbers indicate the youngest age of each respective group (e.g. M_26 is the male 26-35-year-old group). For the box-and-whisker plots, the ratios were calculated as $2^{-\Delta Ct} \times 100$, and the results are presented on a log10 scale. The upper and lower limits of the boxes and the lines inside the boxes indicate the 75th and 25th percentiles and the average, respectively. The upper and lower horizontal bars denote the 90th and 10th percentiles, respectively. The points indicate assay values located outside 80% of the data.

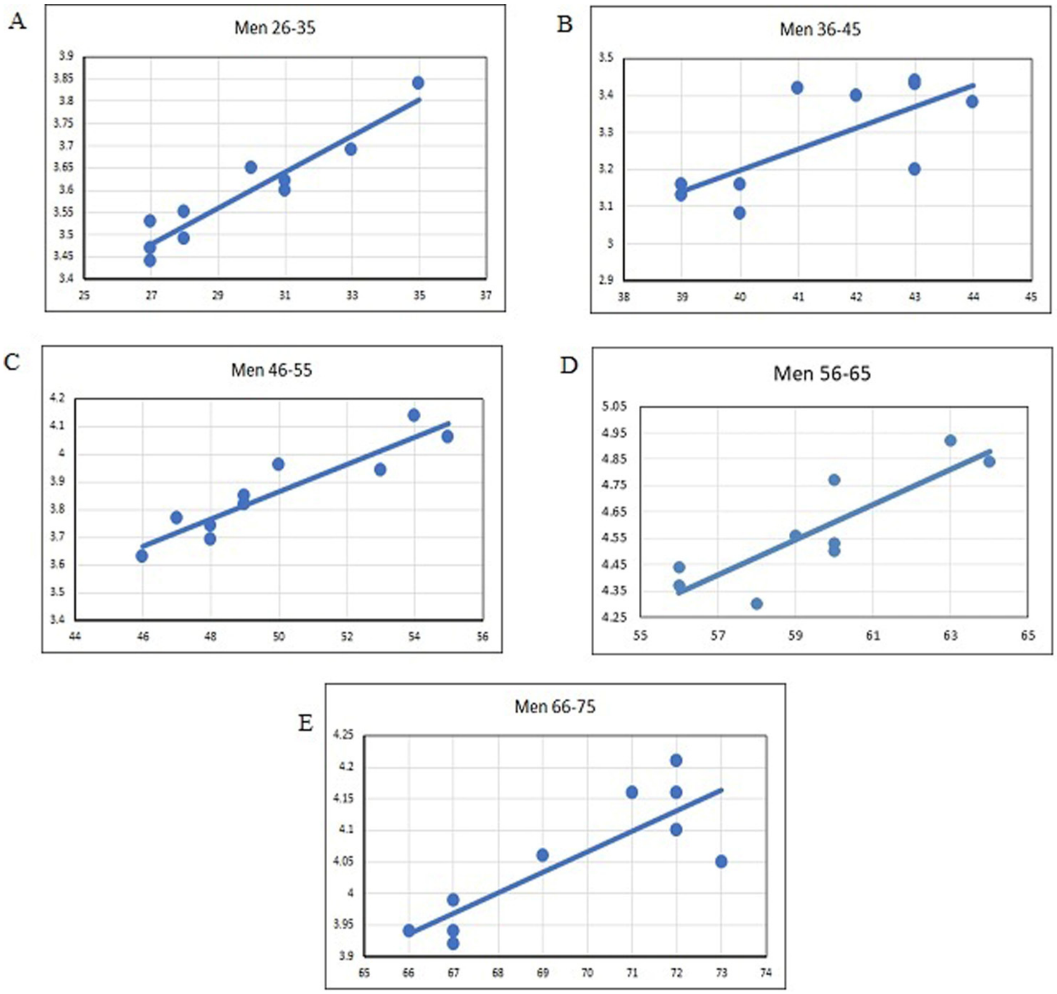

Figure S3. Spearman correlations of the miRNA pair combinations (from Table 5) with subject ages in each of the 10 male groups. Correlation coefficients, P-values and residual standard deviation (RSD) values are indicated in Table 3. X axis: age of subjects; Y axis: the average of the pair ratios in each respective combination, calculated as described in the legend for Figure S2.

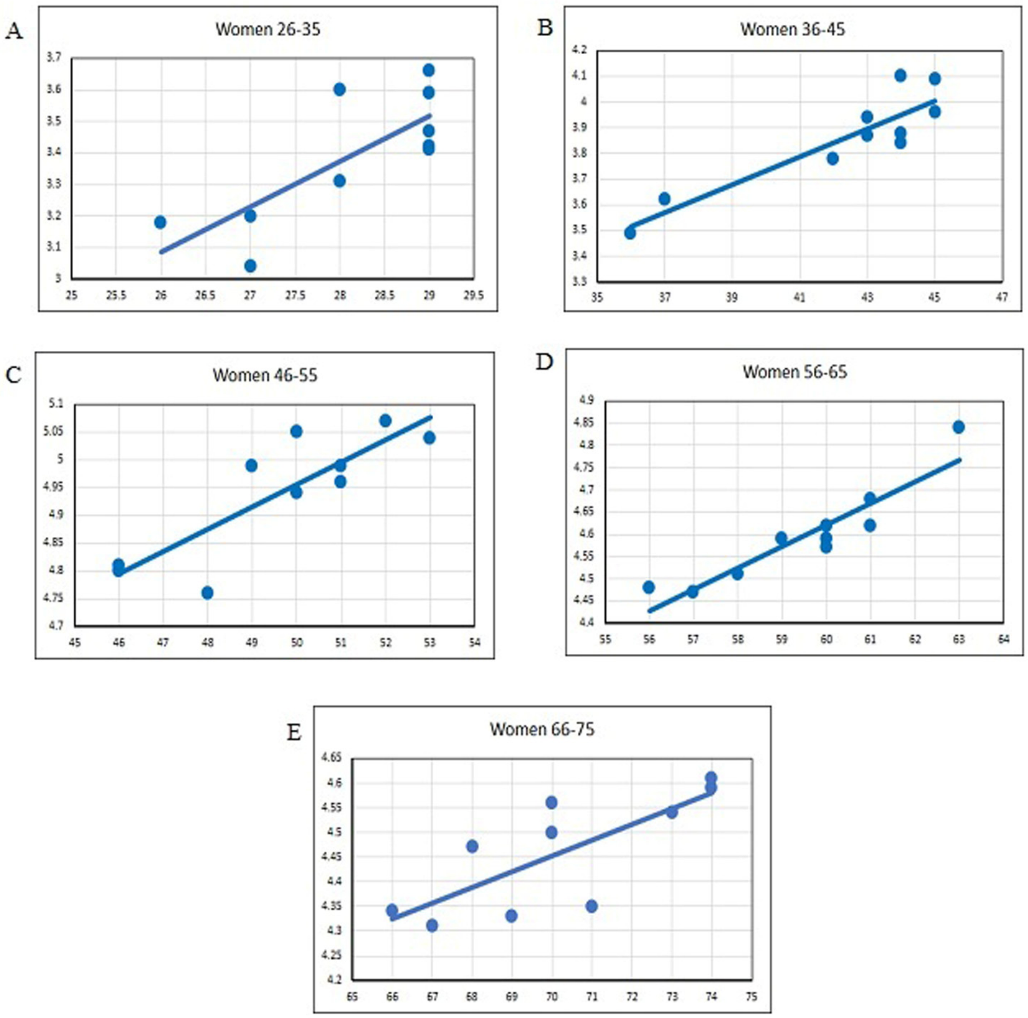

Figure S3. (cont)**. Spearman correlations of the miRNA pair combinations (from Table 5) with subject ages in each of the 10 female groups.** Correlation coefficients, P-values and residual standard deviation (RSD) values are presented in Table 3. X axis: age of subjects; Y axis: the average of the pair ratios in each respective combination, calculated as described in the legend for Figure S2.
