# Supplementary material for: Age- and sex-dependent changes in levels of circulating brain-enriched microRNAs during normal aging
Source: Aging (Albany NY). 2018 Oct 31;10(10):3017–41. doi: 10.18632/aging.101613 (PMC6224262; doi:10.18632/aging.101613)
Supplement: Supplementary Tables [file aging-10-101613-s002.pdf]

## SUPPLEMENTARY TABLES

**Table S1. Heat map of Ct differences in the miRNAs tested in the first set of experiments and the list of miRNAs selected for the second set of experiments.**

| | Young males - Young females | Young males - Old males | Young females - Old females | Old males - Old females | Selected for use in Study 2 |
|---|---|---|---|---|---|
| miR-7 | -0.33 | 0.57 | 0.23 | -0.66 | miR-7 |
| let-7e | 0.25 | 0.70 | 0.20 | -0.26 | let-7e |
| miR-107 | 0.01 | 0.29 | 0.15 | -0.13 | |
| miR-127 | 0.09 | -0.04 | -0.97 | -0.83 | miR-127 |
| miR-128a | -0.06 | 0.19 | 0.25 | 0.00 | |
| miR-132 | 0.34 | 0.74 | 0.35 | -0.05 | miR-132 |
| miR-135a | 0.39 | 1.49 | 0.53 | -0.57 | miR-135a |
| miR-16 | -0.11 | 0.40 | 0.22 | -0.29 | |
| miR-181a | 0.49 | 0.97 | 0.81 | 0.32 | miR-181a |
| miR-182 | -0.13 | 0.72 | 0.85 | -0.01 | miR-182 |
| miR-195 | 0.00 | 0.61 | 0.61 | -0.01 | miR-195 |
| miR-200a | 0.10 | -0.24 | 0.48 | 0.82 | |
| miR-323-3p | 0.13 | 0.07 | -0.67 | -0.61 | miR-323-3p |
| miR-335 | 0.08 | 0.17 | 0.35 | 0.26 | |
| miR-338-3p | -0.15 | -0.24 | 0.15 | 0.24 | |
| miR-370 | 1.44 | 0.61 | -1.60 | -0.77 | miR-370 |
| miR-375 | -0.39 | -0.16 | 1.43 | 1.20 | miR-375 |
| miR-382 | 0.33 | 1.03 | 0.39 | -0.31 | miR-382 |
| miR-410 | 0.21 | 0.51 | -0.18 | -0.48 | |
| miR-411 | 0.71 | 0.65 | -0.36 | -0.30 | miR-411 |
| miR-433 | 0.24 | 0.05 | -0.91 | -0.73 | miR-433 |
| miR-485-5p | 0.48 | 0.33 | -0.30 | -0.15 | |
| miR-487b | 0.38 | 0.60 | -0.17 | -0.39 | miR-487b |
| miR-874 | 0.10 | 0.44 | 0.61 | 0.28 | miR-874 |
| miR-9 | 0.67 | 0.64 | 0.24 | 0.27 | |
| miR-9* | 0.32 | -0.31 | -0.08 | 0.55 | |
| miR-99a | 0.38 | 0.98 | 0.82 | 0.23 | miR-99a |
| miR-134 | 0.25 | 0.49 | -0.81 | -1.05 | miR-134 |
| miR-451 | -0.17 | 0.27 | 0.23 | -0.21 | |
| miR-491 | -0.03 | 0.28 | 0.06 | -0.24 | miR-491 |

**Table S2. Heat map of the correlational data presented in Figure S1.**

| | Age | miR-127 | miR-134 | miR-323-3p | miR-370 | miR-382 | miR-411 | miR-433 | miR-487b |
|---|---|---|---|---|---|---|---|---|---|
| Male | 29.7 ± 2.79 | -0.03 | -0.19 | -0.17 | -0.37 | -0.10 | 0.12 | -0.19 | -0.09 |
| | 41.4 ± 1.84 | 0.20 | 0.09 | 0.03 | -0.25 | 0.28 | 0.17 | 0.13 | 0.05 |
| | 49.8 ± 3.22 | -0.69 | -0.72 | -0.40 | -0.63 | -0.50 | -0.61 | -0.60 | -0.58 |
| | 60.4 ± 3.72 | -0.32 | -0.29 | -0.21 | -0.12 | -0.40 | -0.32 | -0.25 | -0.39 |
| | 69.6 ± 2.67 | 0.59 | 0.62 | 0.54 | 0.48 | 0.50 | 0.29 | 0.46 | 0.52 |
| Female | 28.1 ± 1.1 | -0.34 | -0.51 | -0.38 | -0.43 | -0.45 | -0.05 | -0.27 | -0.42 |
| | 42.4 ± 3.31 | 0.31 | 0.44 | 0.36 | 0.33 | 0.41 | 0.18 | 0.40 | 0.37 |
| | 49.6 ± 2.37 | -0.06 | 0.07 | 0.16 | -0.44 | 0.01 | -0.30 | -0.04 | 0.01 |
| | 59.6 ± 2.07 | 0.10 | -0.01 | 0.10 | -0.34 | 0.06 | 0.08 | 0.09 | 0.04 |
| | 70.2 ± 2.82 | -0.37 | -0.28 | -0.36 | -0.31 | -0.13 | -0.55 | -0.35 | -0.44 |
